# Ultrasound examination of the normal fetal duodenum

**DOI:** 10.3389/fsurg.2022.940376

**Published:** 2023-01-06

**Authors:** Huibin Xiao, Hongbo Li, Xiaozhu Chen, Xiaoyan Lin, Xiaoqin Liang, Huoyong Jiang, Hongchan Wang, Wenyue Lu, Fengrong Li, Zhenping He

**Affiliations:** ^1^ Department of Ultrasound, People's Hospital of Longhua, Shenzhen, China; ^2^ Community Health Service Center, People's Hospital of Longhua, Shenzhen, China

**Keywords:** ultrasound imaging, pylorus, fetal duodenum, normal duodenum, duodenal bulb

## Abstract

**Objective:**

To use the fetal pylorus as a reference point to conveniently display the normal fetal duodenum by ultrasound.

**Methods:**

This study was designed in cross-section. A total of 450 healthy singleton pregnant women at 19–39 weeks of gestation who underwent prenatal screening at our hospital from January 2019 to February 2020 were selected. They were divided into three groups according to gestational weeks: the 19–23 gestational weeks group, 29–32 gestational weeks group, and 34–39 gestational weeks group. The duodenal bulb was identified. Its movement and course were continuously and dynamically observed. The descending part of the duodenum was identified, and the duodenal course was traced.

**Results:**

The fluid-filled in the fetal duodenum was discontinuous. The overall detection rates of the duodenum in the 19–23 gestational weeks group, 29–32 gestational weeks group, and the 34–39 gestational weeks group were 82.2%, 26.2%, and 13.8%, respectively. The detection rates of the bulbar, descending, horizontal, and ascending parts of the duodenum were 94.4%, 58.2%, 58.0%, and 52.0%, respectively. The anatomical structures of the duodenum as a whole and the pancreas were most easily recognized in the 19–23 gestational weeks group; while in the 34–39 gestational weeks group, the bulbar part had a maximum detection rate of 98.8%, and it had the longest filling time and the shortest examination time.

**Conclusion:**

The pylorus is an ideal starting point for tracing the fetal duodenum. The overall detection rate of the fetal duodenum decreases with gestational age. The duodenal bulb is the most easily detected site.

## Introduction

Duodenal atresia and stenosis are the most common intestinal obstruction in neonates, accounting for 37.0%–49.0% of small intestinal atresia (1). The etiology of this condition is unknown. These can occur in any part of the duodenum, most commonly in the second segment of the duodenum. The characteristic ultrasound manifestation of duodenal atresia and stenosis in the fetus is a significant dilatation of the proximal stomach and duodenum, called the “double bubble sign”. The “double bubble sign” is usually observed in the late second trimester or early third trimesters of pregnancy, making it difficult to diagnose during the early stages of fetal development (1, 2). There are few studies analyzing the features of the normal fetal duodenum.

Ultrasound is ideal for examining the fetal duodenum due to its ability to assess the duodenum dynamically and in real time, the absence of ionizing radiation, and its high-resolution rendering. Transabdominal ultrasound has been used to examine the fetal duodenum, demonstrating that ultrasonography is a reliable tool for fetal duodenum (3, 4).

This study was conducted to investigate the significance of the normal fetal duodenum by serially following dynamic ultrasound images of 450 fluid-filled duodenum cases. The purpose of this study was to use the fetal pylorus as a reference point to conveniently display the normal fetal duodenum by ultrasound.

## Materials and methods

### General information

This study was a cross-sectional design. All pregnant women signed the informed consent form for a prenatal ultrasound examination. Informed consent was waived by the Institutional Review Board of Shenzhen Longhua Hospital, due to the retrospective, non-interventional nature of the study. A total of 450 women with a normal singleton pregnancy who underwent prenatal Class I and II fetal screening in our hospital from January 2019 to February 2020 were selected. The pregnant mothers had no previous history of hypertension, diabetes, genetic diseases, and radiation or drug exposure during pregnancy. The pregnant women were divided into three groups according to several periods of routine ultrasound examination throughout the gestational weeks: the 19–23 gestational weeks group, 29–32 gestational weeks group, and 34–39 gestational weeks group. Fetuses were screened for Down's syndrome or congenital anomalies.

### Instruments and methods

Using GE Voluson E8 and GE E10 color Doppler ultrasound diagnostic instruments with a probe frequency of 2.0–5.0 MHz, pregnant women enrolled in the study were instructed to lie in the supine or lateral position. In addition to the routine systematic ultrasound examinations according to the requirements of the prenatal ultrasound examination guidelines (5), observation of the fetal duodenum and corresponding ultrasound imaging features were included. The lateral decubitus or supine positions of the fetus were preferred to display the gastric bubble in the abdominal transverse section. After which, the rightmost side of the gastric bubble (i.e., the pylorus) was observed. The probe was moved slightly upward and downward to observe whether there was an intestinal canal with echoes of fluid filling anterior to, on the right side, and inferoposterior to the pancreas.

After assessing the pylorus, we confirmed whether it was continuous with the gastric bubble based on the fluid filling in each segment of the duodenum. The course of the fetal duodenum was continuously and dynamically tracked. After identifying the fetal duodenum, the following contents were recorded: (1) the examination time of the pylorus and duodenum; (2) the detection rate of the pylorus and each segment of the duodenum; (3) the ultrasound characteristics of the pylorus and duodenum; and (4) pregnancy outcomes of all fetuses during follow-up.

### Statistical analysis

SPSS 19.0 statistical software was used to compare the detection rate and examination time of each segment of the pylorus and duodenum. The data for quantitative information were recorded as mean standard deviation (*x̅ *± *s*). The frequency distribution of qualitative information was expressed as a percentage.

## Results

### Patient demographics

The women were all of Asian descent. Their ages ranged from 19 to 41 years (28.6 ± 3.8 years) and most were within the normal BMI range (18–24 kg/m^2^). They were all within 0–2 pregnancies and conceived naturally. Most fetuses were in the 19–23 gestational weeks group (225 fetuses, 21.7 ± 0.5 weeks), followed by the 29–32 gestational weeks group (145 fetuses, 29.9 ± 0.8 weeks), and finally the 34–39 gestational weeks group (80 fetuses, 36 ± 1.4 weeks). All fetuses included in this study showed a low risk for Down's syndrome screening and were structurally normal according to ultrasound examination. No congenital anomalies were noted in all fetuses. The absence of significant gastrointestinal diseases 1 month after birth was considered normal.

### Ultrasound findings

We examined 225 women at 19–23 weeks gestation, 145 women at 29–32 weeks gestation, and 80 women at 34–39 weeks gestation. In the 19–23 weeks gestation group, the majority of pregnancies (67.1%) were between 21 and 22 weeks gestation; in the 29–32 weeks gestation group, most of the pregnancies (60.7%) were between 29 and 30 weeks gestation; and in the 34–39 weeks gestation group, the majority of pregnancies (86.3%) were between 35 and 39 weeks gestation.

The total pylorus detection rate for all participants in this study was 99.1%. The greater the gestational weeks, the shorter the examination time. The smaller the gestational weeks, the higher the overall detection rate of the duodenum. The detection rate of each segment of the duodenum in different gestational weeks was different. The duodenal bulb was the easiest to track, with a detection rate of 94.4%. The descending part and horizontal part of the duodenum showed the highest detection rates in the 19–23 gestational weeks group (86.2% and 89.3%). The detection rates of the descending part and horizontal part of the duodenum in the 29–32 gestational weeks group were significantly lower than those in the 19–23 gestational weeks group. The horizontal part and ascending part of the duodenum were not observed in any of the cases in the 35–39 gestational weeks (Table 1).

**Table 1:** Display rate of pylorus and each segment of the duodenum in the three study groups.

Position	19–23 Weeks (*n* = 225)		29–32 Weeks (*n* = 145)		34–39 Weeks (*n* = 80)		Total (*n* = 450)	
	NO.	%	NO.	%	NO.	%	NO.	%
Pylorus	221	98.2	145	100.0	80	100.0	446	99.1
DB	206	91.6	140	96.6	79	98.8	425	94.4
DD	194	86.2	57	39.3	23	28.8	274	60.9
HD	201	89.3	45	31.0	15^a^	18.8	261	58.0
AD	185	82.2	38	26.2	11^a^	13.8	234	52.0

DB, duodenal bulb; DD, descending part of the duodenum; HD, horizontal part of the duodenum; AD, ascending part of the duodenum.

^a^ These cases were in the range of 34w−34w + 6.

In the 34–39 gestational weeks group, the time taken for examining the pylorus and bulb was the shortest, and duodenal bulb was synchronously displayed in a filling state. The examination time of 73.8% (59/80) of the fetuses was <1 min, that of 92.5% (78/80) of the fetuses was <5 min, and the longest examination time was about 10 min (Table 2).

**Table 2:** The examination time of the pylorus and each segment of the duodenum in the three study groups (minutes).

Position	19–23 Weeks (*n* = 225)	29–32 Weeks (*n* = 145)	34–39 Weeks (*n* = 80)
Pylorus	2.4 ± 1.5	1.3 ± 1.1	0.7 ± 0.8
DB	4.7 ± 3.2	2.4 ± 1.9	1.1 ± 1.6
DD	7.5 ± 5.7	5.5 ± 4.0	4.1 ± 2.2
HD	5.3 ± 3.5	5.0 ± 2.5	4.5 ± 2.9
AD	8.4 ± 5.3	6.1 ± 2.3	4.6 ± 2.4

DB, duodenal bulb; DD, descending part of the duodenum; HD, horizontal part of the duodenum; AD, ascending part of the duodenum.

As a structure connecting the fetal gastric bubble and duodenum, the pylorus had the highest detection rate and the shortest examination time in the three groups, so the pylorus can be used as an ideal starting point for tracking the course of the duodenum (6).

### Ultrasound features of the fetal pylorus

**Figure 1:**
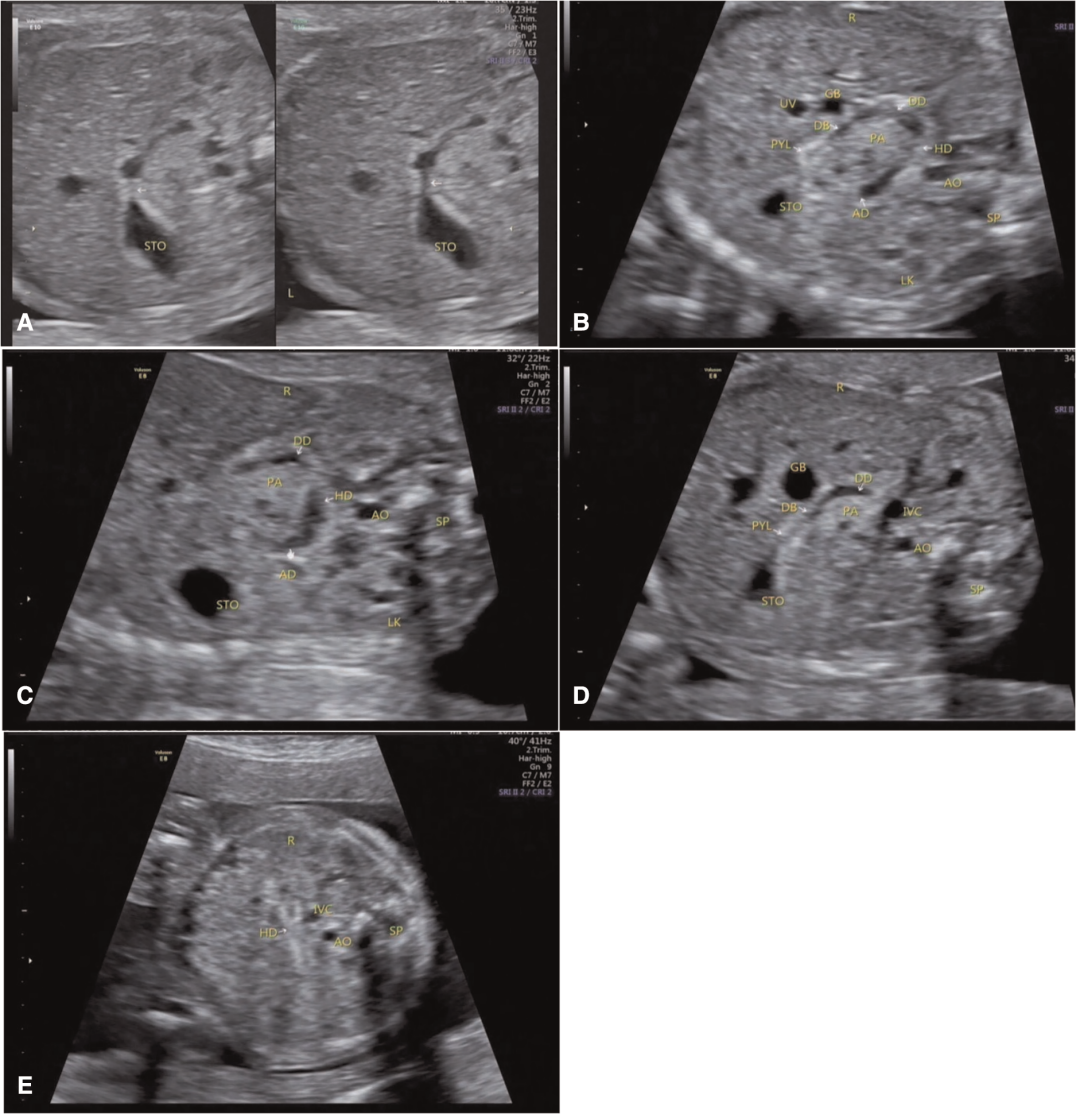
Duodenal images were displayed at 22 gestational weeks. (**A**) The pylorus was in open and closed states, and is hyperechoic. (**B**) Whole-course simultaneous display of the stomach, pylorus, and duodenum. (**C**) The filling state of the descending, horizontal, and ascending parts of the duodenum and their relationships with the position of the head of pancreas. (**D**) The descending part of the duodenum and its positional relationship with the head of the pancreas. (**E**) The collapsed state of the horizontal part of the duodenum. STO, stomach; PYL, pylorus; DB, duodenal bulb; DD, descending part of the duodenum; HD, horizontal part of the duodenum; AD, ascending part of the duodenum; AO, abdominal aorta; IVC, inferior vena cava; LK, left kidney; SP, spine; GB, gall bladder; PA, pancreas; UV, umbilical vein; R, right side of the fetus; L, left side of the fetus.

**Figure 2:**
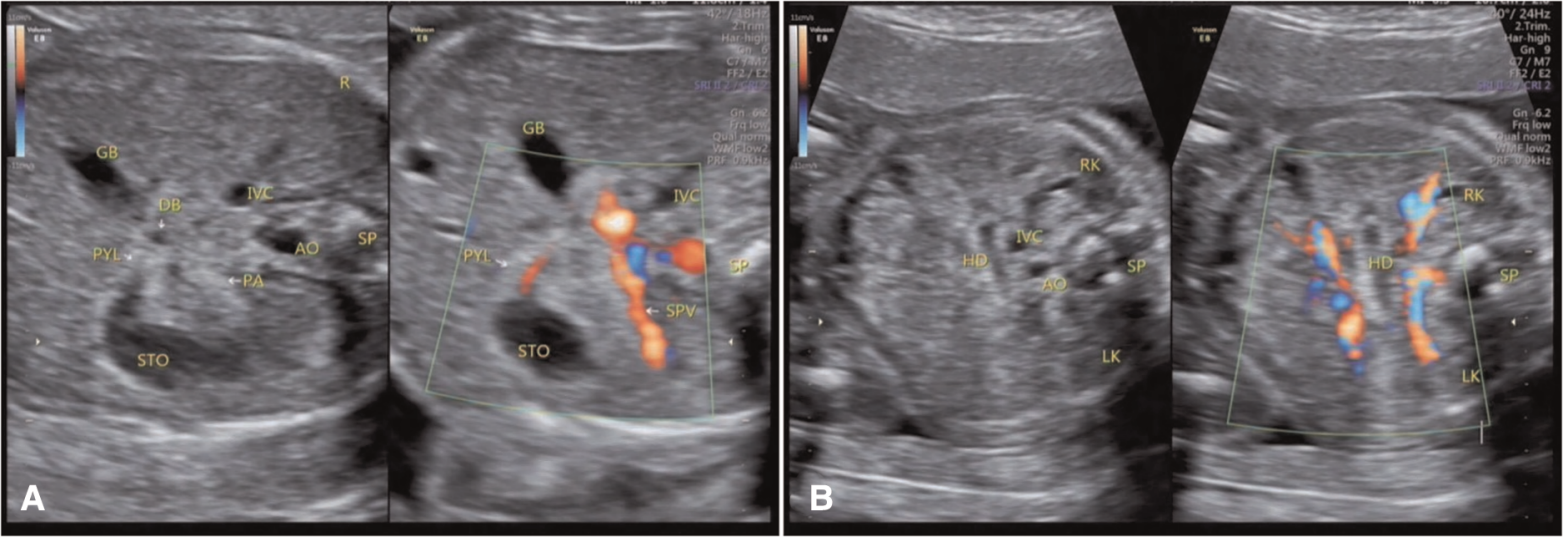
Color Doppler flow imaging of duodenum at 22 gestational weeks. (**A**) The moment the gastric contents passed through the pylorus. (**B**) The filling state of the horizontal part of the duodenum. STO, stomach; PYL, pylorus; DB, duodenal bulb; HD, horizontal part of the duodenum; AO, abdominal aorta; IVC, inferior vena cava; SPV, spleen vena; GB, gall bladder; PA, pancreas, RK, right kidney; LK, left kidney; SP, spine; R, right side of the fetus.

**Figure 3:**
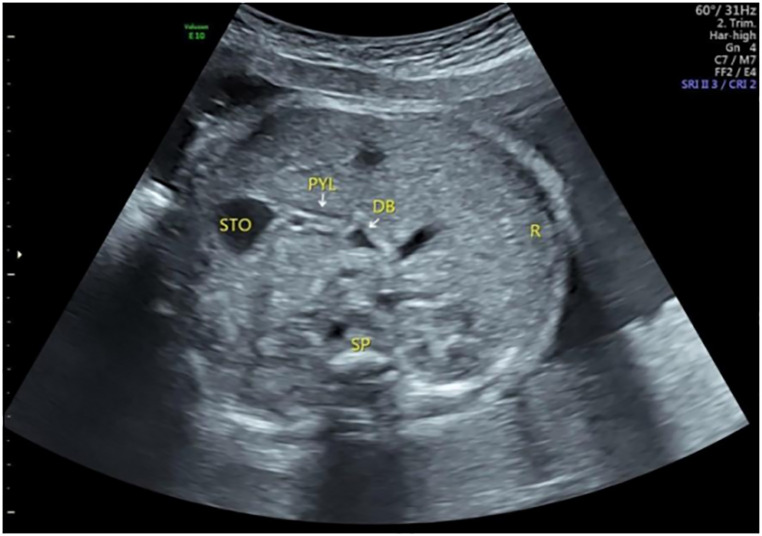
The pylorus and duodenal bulb at 30 gestational weeks. The pylorus was composed of three and five layers, mainly composed of hyperechoic mucosa and hypoechoic muscular layer. STO, stomach; PYL, pylorus; DB, duodenal bulb; SP, spine; R, right side of the fetus.

When the pylorus is in a closed state, a five-layer structure with high-low-high-low-high echo intensity is displayed from the outside to the inside. In the open state, the single-layer lumen wall presented a three-layer structure with high-low-high echo intensity centrifugally, all of which were mainly with high echo intensity in the internal layer. At relatively small gestational weeks, only high echo intensity was presented (7) (Figures 1A, 2A, 3).

### Ultrasound features of fetal duodenum

#### Duodenal wall

The duodenal wall of the fetuses showed high echo intensity. When there is fluid filling in the duodenum, this state is an important ultrasound image feature to confirm the course of the duodenum. High echo intensity indicates the lumen wall, while the filled fluid is anechoic. Under dynamic tracking observation along the long axis of the duodenum, the fluid echoes in the intestinal canal move downward with the movement of the intestinal wall, and the course of the duodenum could be observed when the fluid moves downward (Figures 1B,C).

#### Pylorus

**Figure 4:**
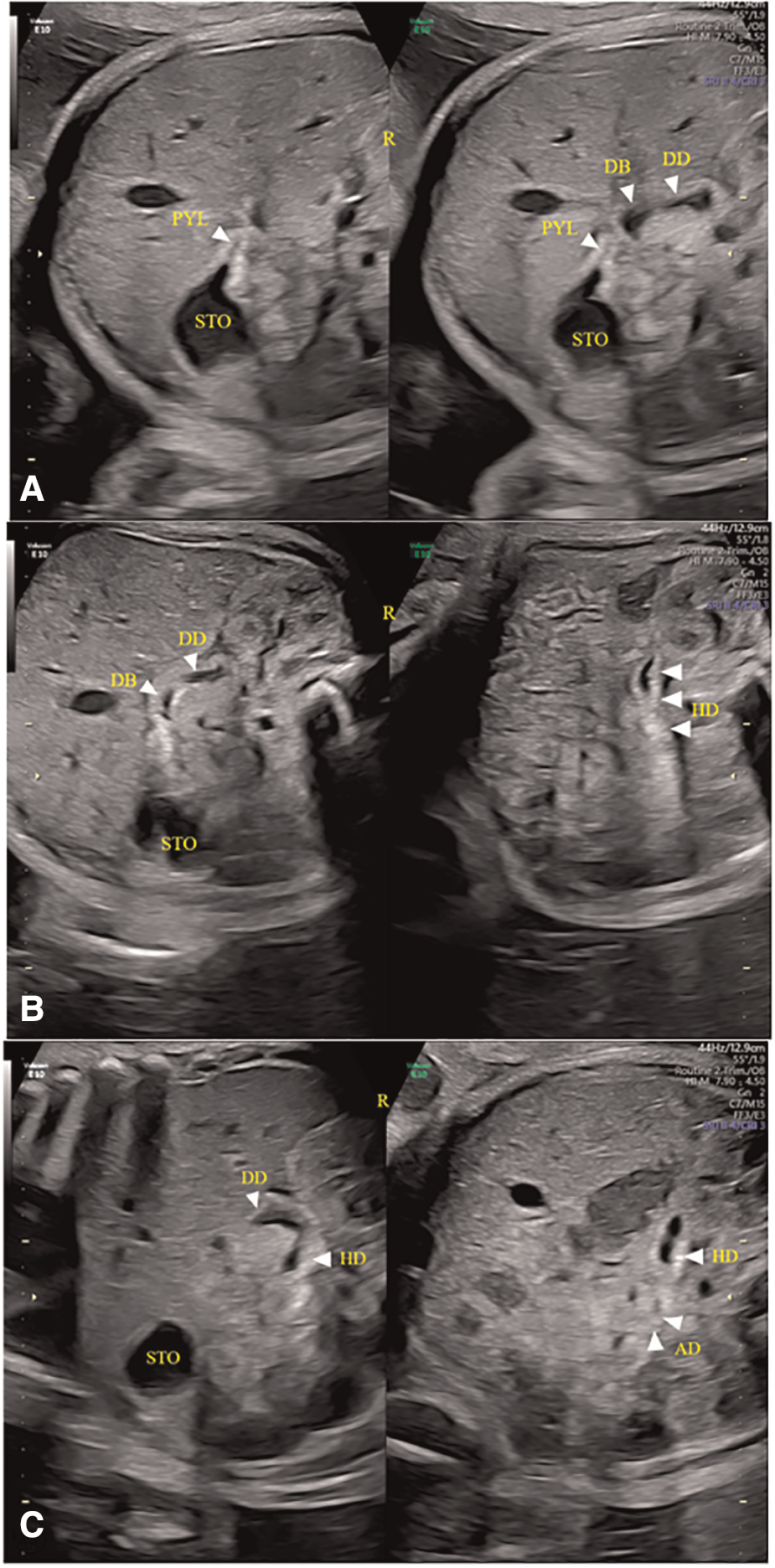
Duodenal images were displayed at 31 gestational weeks. (**A–C**) The wall of the duodenal canal was thicker, wider, and slightly curved than that in 19–23 Weeks of gestation. STO, stomach; PYL, pylorus; DB, duodenal bulb; DD, descending part of the duodenum; HD, horizontal part of the duodenum; R, right side of the fetus.

When the fetal duodenum is in a collapsed state, the bulb can be seen on the right side of the pylorus, showing a relatively wide long strip-shaped high echo intensity, with unclear or clear boundaries and poor continuity, which makes it difficult to distinguish the “C” structure surrounding the head of the pancreas (Figures 1B,D). In this state, ultrasound identification is difficult, and it needs to be confirmed in the filling state. In some fetuses (88/225) in the 19–23 gestational weeks group, the horizontal part of the duodenum anterior to the spine could be directly observed in the transverse or coronal plane of the abdomen (Figures 1E, 2B), and its ultrasound feature was a relatively wide band-shaped high echo intensity (Table 3). As the gestational week increases, the internal diameter of the duodenum widens, the thickness of the wall thickens, and the shape changes from flat to curved. (Figures 4A–C).

**Table 3:** Comparison of the display of various duodenal parts in the collapsed and filling states in the 19–23 gestational weeks group.

19–23 Weeks (*n* = 225)	Collapsed duo^a^	Fluid-fifilled duo	Proportion
	NO.	NO.	%
DB	23	206	11.2
DD	15	194	7.7
HD	88	201	43.8
AD	0	185	0.0

DB, duodenal bulb; DD, descending part of the duodenum; HD, horizontal part of the duodenum; AD, ascending part of the duodenum.

^a^ The duodenum in a collapsed state means that the duodenum can be identified without filling, but it needs to be confirmed after filling.

#### Duodenal bulb

**Figure 5:**
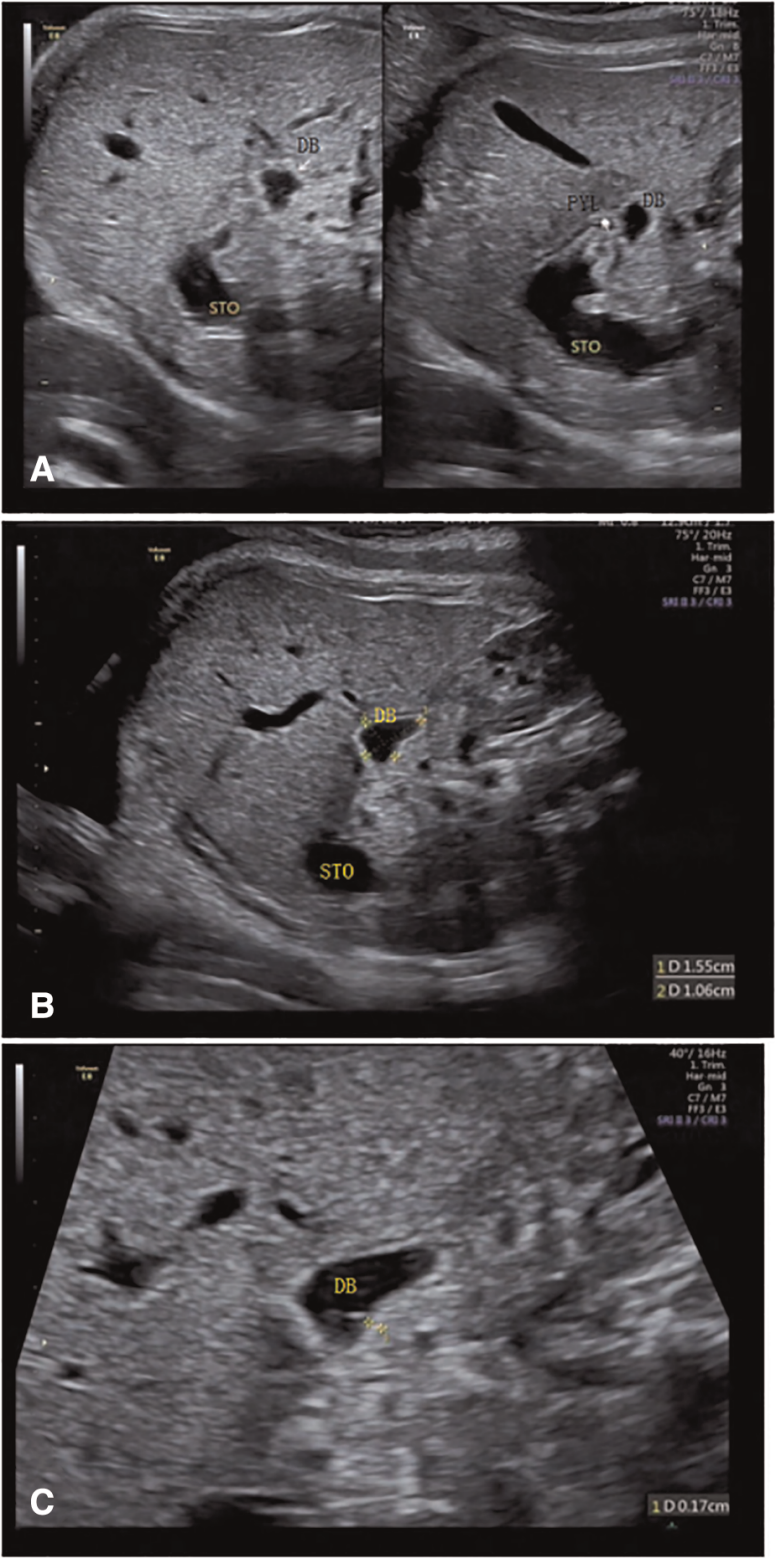
The duodenal bulb was large when filled with fluid at 38 gestational weeks, and dynamic observation showed significant changes. (**A, B**) After being filled in by fetal stomach contents, the volume of the duodenal bulb increases significantly. (**C**) Duodenal canal wall thickness were measured after local magnification. STO, stomach; DB, duodenal bulb.

The normal fetal duodenum does not have a wide internal diameter and is filled intermittently and for a short period of time, which may decrease significantly or disappear during dynamic observation. In a few fetuses in the 34–39 gestational weeks group, when there was fluid filling in the duodenal bulb, the duodenal bulb had a maximum length of 16 mm and a maximum width of 11 mm. Because of its large scope, similar to an oval shape, the duodenal bulb together with the gastric bubble may present a false “double bubble sign” (Figure 5A-C). Its ultrasound feature is that the gastric bubble is significantly larger than the duodenal bulb, the intestinal wall is thicker with low tension, and its size may decrease or it disappears under dynamic observation.

## Discussion

We aimed to demonstrate the ultrasound examination findings on normal fetuses to explore the clinical value of this imaging modality in identifying the duodenal tract. We revealed that the fetal pylorus may be used as a reference point to display the normal fetal duodenum by ultrasound. Further, the detection rate of the whole duodenum decreases with the increase of gestational week, and the examination time increases.

The duodenum is an organ between the stomach and the jejunum. Its shape resembles the letter “C”. It surrounds the head of the pancreas and can be divided into four parts, i.e., the bulbar part, the descending part, the horizontal part, and the ascending part of the duodenum. The duodenum is also a long, tubular, soft tissue structure, which is usually collapsed. It cannot be easily distinguished from the surrounding tissue structures on ultrasound images. When the “double bubble sign” is identified, it must be determined whether it communicates with the normal gastric bubble. If the “double bubble sign” is not shown during the early stages of fetal development, scanning to display the entire normal duodenum is necessary. The filling state of the duodenum lasts for a short time, during which it is mainly collapsed.

### Research status of the duodenum

Desdicioglu (8), Gu (9), and Zhong et al. (10) collected data on the morphology of the duodenum, namely the height, width, length, and diameter, as well as the vascular morphology of each layer autopsy findings. Koyuncu et al. (11) explored the location of the pylorus and its positional relationship with adjacent organs and found that the thickness of the pylorus and duodenal muscular layer had a significant relationship with gestational age. At present, there is no relevant ultrasound study on the normal duodenum during the fetal period. In this study, the demonstration of the fetal duodenal segments was most comprehensive in the 19–23 gestational weeks group, and the continuity of each segment could be directly displayed when the intestinal canal was filled. The inner diameter of each segment changed at any time due to intestinal movement. Although accurate data could not be obtained, no significant difference in the inner diameter of each segment could be visually observed. The anatomic relationship of the duodenum with the peripheral tissues could also be shown, especially its spatial relationship with the pancreas.

**Figure 6:**
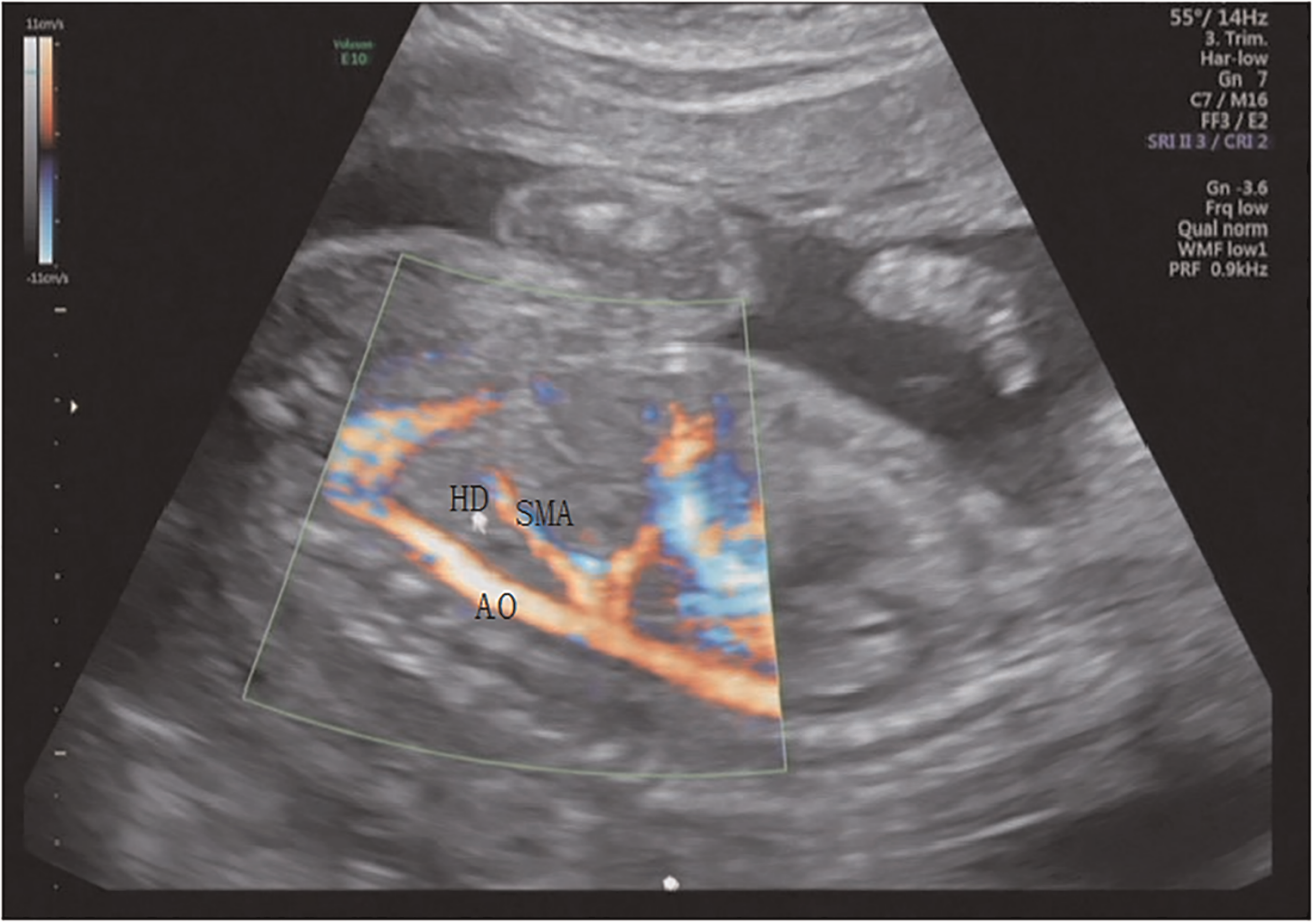
The positional relationships of the horizontal cross-section of the duodenum with the abdominal aorta and superior mesenteric artery in the sagittal section at 20 gestational weeks. HD, horizontal part of the duodenum; AO, abdominal aorta; SMA, superior mesenteric artery.

Therefore, this study predicts that obstructive changes can be ruled out when fluid filling from the duodenal bulb to the ascending part and intestinal movement can be observed, without significant difference in intestinal canal diameter. When combined with color Doppler ultrasonography, a relatively satisfactory relationship between the horizontal part of the duodenum and the superior mesenteric artery can be obtained for a few fetuses (12, 13) (Figure 6). In the 19–23 gestational weeks group, the spatial structure and course of each segment were most easily observed, but the time required was the longest, and the effective observation time was short, which might be related to infrequent stimulation due to the lack of food in the intestinal canal during this period. This needs to be verified in future studies whether the mechanism of intestinal movement in the fetal period is different from that in neonates.

### Correlation of the detection rates of the fetal pylorus and duodenum with gestational age

The detection rate of the whole duodenum decreases as gestational week increases, and the examination time increases. In this study, the detection rate was the highest in the 19–23 gestational weeks group, and it was easy to distinguish the duodenum from the pancreas, an important surrounding tissue. The detection rates of the pylorus and duodenal bulb increased with increasing gestational age, while the examination time was significantly reduced. The detection rate of the duodenal bulb increased significantly. The greater the gestational weeks, the lower the influences of the fetal position, amniotic fluid volume, and abdominal wall thickness of the pregnant women on the pylorus. The larger the scope and the longer the duration of fluid filling in the bulb, the better the structural relationships of the bulb and pylorus with the gastric bubble can be shown.

In our experience, external factors affecting fetal duodenal ultrasonography include: fetal position, high BMI, abnormal amniotic fluid, frequent fetal movements, and frequent breathing. Among them, fetal position and high BMI are the most important factors. The detection rates of the descending part, horizontal part, and ascending part in the third trimester decreased significantly with increasing gestational weeks. After 35 weeks, the horizontal part and ascending part of the duodenum can be shown at a filling state theoretically, but not in this study. In this study, it was speculated that it might be related to the fact that at higher gestational weeks, the amniotic fluid volume decreased and the intestinal contents (14) increased, resulting in the changes in their echoes, thus, making it difficult to distinguish from the surrounding structures.

### Research limitations and prospects

In this study, with the pylorus as the starting point, there was no difficulty in identifying the duodenum through continuous dynamic tracking when there were echoes of fluid filling in the duodenum. Particularly, during movements, the relationship between the duodenum and the surrounding organs could be confirmed and observed, which was of great significance to rule out obstructive lesions in the duodenum (15). However, it is difficult to show the continuity of the duodenum in the collapsed state.

There was alternating filling and collapsed states of the duodenal canal, predominantly occurring in the collapsed state. Only a few fetuses (2.2%) had a long overall filling time, and the whole duodenum could be observed with a slight lateral movement of the probe on one section (Video 1, 2 and 3). Most fetuses had a short filling time of the intestinal canal, so it was difficult to sufficient information during the short time available. This posed a challenge for the dynamic observation of fetal duodenal continuity in this study. We recommend that ultrasound be performed only in fetuses suspected of having duodenal obstruction, and that ultrasound be performed preferably in the middle of pregnancy.

## Conclusion

In conclusion, this study has preliminarily verified the feasibility of ultrasound examination of the normal duodenum in the fetal period, which will contribute to the diagnosis of diseases such as early duodenal obstruction, thereby providing more examination options for high-risk pregnant women during the early stages of pregnancy (16, 17), as well as more time for early clinical treatment and intervention.

## Data Availability

The original contributions presented in the study are included in the article/Supplementary Material, further inquiries can be directed to the corresponding author/s.
